# Trends and geographical variations in outpatient antimicrobial consumption in Ireland in relation to socio-economic deprivation

**DOI:** 10.1016/j.heliyon.2024.e37563

**Published:** 2024-09-10

**Authors:** Nathaly Garzón-Orjuela, Doaa Amin, Ajay Oza, Ricardo Segurado, Akke Vellinga

**Affiliations:** a CARA, School of Public Health, Physiotherapy and Sports Science, University College Dublin, D04 C1P1, Ireland; b Health Protection Surveillance Centre (HPSC), Dublin, D01 A4A3, Ireland; c School of Public Health, Physiotherapy and Sports Science, University College Dublin, D04 C1P1, Ireland

**Keywords:** Antimicrobial consumption, Anti-bacterial agents, Trends, Multilevel modelling, Social deprivation, Socioeconomic factors, Geographic variations, Spatial autocorrelation

## Abstract

**Background:**

Different factors have been associated with changes in antimicrobial consumption rates in Ireland, however the relationship between socio-economic deprivation and antimicrobial consumption has not been explored. The presented ecological analysis explores the temporal and geographical variation in outpatient antimicrobial consumption and socio-economic deprivation in Ireland from January 2015 to March 2022.

**Method:**

Deprivation index (DI) was used as a socio-economic proxy. A multilevel mixed model was applied to explore temporal variation and analyse the longitudinal antimicrobial consumption (DID) in relation to DI. Furthermore, maps were generated based on antimicrobial consumption rates, and spatial autocorrelation analyses were carried out to study geographical variation in antimicrobial consumption rates.

**Results:**

The antimicrobial consumption rates per month varied from 26.2 DID (January 2015) to 22.1 DID (March 2022) showing an overall reduction of 16 %. Overall, total antimicrobial consumption in the multilevel model showed a consistent correlation with higher DI score (6.6 (95%CI 3.9 to 9.3)), and winter season (3.6 (95%CI 3.2 to 3.9)). In contrast, before COVID-19 showed significant lower antimicrobial consumption rates compared to during COVID-19 (−4.0 (95%CI -4.7 to −3.23)). No consistent trends were observed for geographical variation between areas.

**Conclusion:**

Antimicrobial consumption rates decreased from 2015 to 2021 in Ireland. No geographical patterns were observed in antimicrobial consumption rates but associations between deprivation and antimicrobial consumption rates were observed.

## 1 Background

In 2019, antimicrobial resistant infections were associated with nearly 5 million deaths worldwide [1]. Although different strategies to optimise antimicrobial consumption have been implemented in primary and secondary care, antimicrobial resistance (AMR) rates have not decreased [2]. In 2013, the antimicrobial consumption (ATC group J01) in Europe was 21.6, which decreased to 19.4 DDD per 1000 inhabitants in 2022. Contrary to an observed decreasing trend for overall antimicrobial consumption in Europe, there was an increasing trend for the consumption of broad-spectrum penicillins, cephalosporins, macrolides (except erythromycin) and fluoroquinolones in community care [3]. In Ireland, the antimicrobial consumption slightly decreased from 2013 to 2022 (20.0–21.5 DDD per 1000 inhabitants per day) in community care, but this trend was not statistically significant [3]. Furthermore, Ireland showed a slightly higher consumption than the EU/EEA population-weighted mean consumption during this period and an increase in antimicrobial consumption in hospitals, particularly of third generation antimicrobials [3,4].

The variation in antimicrobial consumption is associated with differences in microbiological, societal, cultural and economical factors [5]. Socioeconomic factors such as low education, economic growth and lack of awareness directly impact how fast the emergence of resistance develops [5,6]. An analysis of trends and variations in antimicrobial prescribing rates between practices and geographical areas in England (1998–2007) showed higher prescribing rates in larger practices, rural areas and more deprived areas [7]. In addition, geographic area effects, has had deprivation, had indicated a significant relationship with antimicrobial use [8].

In Ireland, associations have been shown between socio-economic deprivation and hospital admission rates, length of stay, re-admissions, spread of COVID-2019, and benzodiazepine consumption [[9], [10], [11], [12]]. However, the relationship between socio-economic deprivation and antimicrobial consumption has not been explored. The presented ecological study explores the temporal and geographical variation in outpatient antimicrobial consumption and socio-economic deprivation in Ireland from January 2015 to March 2022.

## 2 Methods

### 2.1 Setting

According to the census in 2016, Ireland had a population of 4,761,865 [13]. For this analysis the county (areas) level division was used which defines 33 areas, including two separate local areas for Cork, Galway, Limerick and four local areas for Dublin (appendix 1).

### 2.2 Source of data

Antimicrobial consumption data was provided by the Health Protection and Surveillance Centre (HPSC), based on the monthly community antimicrobial sales by area (January 2015–March 2022). Data included the total outpatient antimicrobial consumption measured in defined daily doses (DDD) and anatomical therapeutic chemical (ATC) group antibacterial agents (J01) for systemic use. In addition, antimicrobial agents were categorised into green (preferred) and red (non-preferred) antimicrobials according to national guidelines for prescribing in the community. This categorisation was introduced in 2016 as part of a national antimicrobial awareness campaign for community prescribers. If a general practitioner (GP) or other community prescriber decides to prescribe an antimicrobial, they are encouraged to prescribe antimicrobials from the green list. Green antimicrobial agents include antimicrobials effective for each condition, with few side effects and less likely to lead to resistant infections compared to red agents [14,15] (appendix 2).

A deprivation index (DI) was used as a socio-economic proxy and openly accessible via the Department of Public Health & Primary Care at Trinity College Dublin [16]. This DI was based on the 2016 national census data and combined information on employment, social class, local authority rented housing and car ownership into an absolute score and decile ranging from 1 to 10 based for each area [16,17]. The absolute score was used because it captured the skewed distribution of deprivation values better than deciles [17]. An increased score reflects increased deprivation [18].

To generate maps, the geometries of 33 areas were extracted from two dataset from Ireland's National Geospatial Data Hub (GeoHive) and merged into one file [19,20]. This data was reprojected into web mercator, and coordinates were extracted from the geometries using Python 3.9.12. The geographical coordinates for all areas can be found in appendix 1.

### 2.3 Outcomes

Antimicrobial consumption (DDD) was standardized using the population-weighted mean by calculating the ratio between the population from the national census and the population reported in Eurostat [21] resulting in DDD per 1,000 inhabitants per day (DID) (antimicrobial consumption rates).

### 2.4 Data analysis

A multilevel mixed model was applied to explore temporal variation and analyse the longitudinal antimicrobial consumption (DID) [22,23]. The model followed a two-level hierarchy for the longitudinal antimicrobial consumption data with repeated measurements of monthly DIDs (87 periods or months from January 2015 to March 2022, as a total of seven years and three months) by the 33 areas (Appendix 1, Appendix 3). Before running the models, the distribution of DID was checked by country and 33 areas (appendix 4). Each model included the variance (random intercept) to adjust for the dependency of the repeated monthly observations within each area. The structure of residual errors (autoregressive process of order 1 (AR(1))) was added to the final multilevel model to improve the model's fit and provide more accurate estimates of the model parameters (the assumptions of homoscedasticity and independence). This structure can be particularly important in longitudinal data with repeated measurements, where the residual errors may be correlated over time [24]. The AR(1) structure was selected based on the time series analysis of the continuous variable (DID) which showed a spike at a lag of 1 and lower spikes for subsequent lags [25]. Each model included absolute deprivation score as well as a variable to mark the COVID-19 pandemic (before and during 2020), a variable for winter, gender and population under 12 years old. The analysis was performed using STATA 16 and R version 4.1.0 statistical software.

To explore geographical variations at area level, maps were generated based on antimicrobial consumption rates (total and before-during COVID-19) and on consumption rates based on the green and red categorisation and deprivation index. In addition, spatial autocorrelation tests (general and local) were carried out to analyse the geographical variation of antimicrobial consumption rates on an area level and explore spatial patterns. The geographical analysis was performed using Python 3.9.12 and Jupyter Notebook.

## 3 Results

**Fig. 1:**
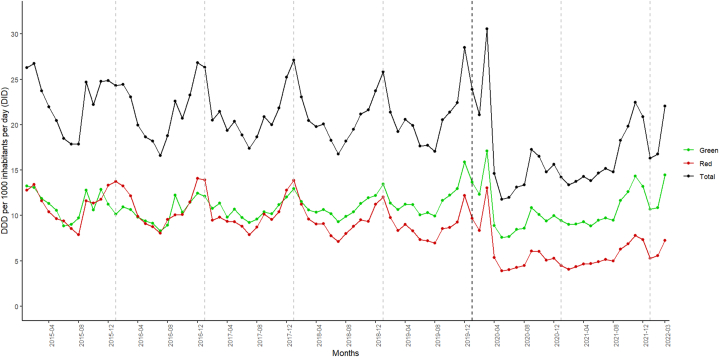
Outpatient consumption of total, green and red antimicrobial use (ATC group J01) in Ireland for 2015 to 2021 by month.

Source: elaborated with data provided by Health Protection and Surveillance Centre based on the antimicrobial sales dataset for Ireland which included the total outpatient antimicrobial consumption measured in defined daily doses (DDD) and anatomical therapeutic chemical (ATC) group antibacterial agents (J01) for systemic use. X-axis: Gray dashed lines show the beginning of each year and black dashed line divide the timeline after and during COVID-19.

In Ireland, the antimicrobial consumption rates per month varied from 23.7 DID in March 2015 to 22.1 DID in March 2022, which was an overall reduction of 7 %. The consumption rates of the green antimicrobial group increased 9 % from 11.8 DID to 14.4 DID (March 2015 to March 2022, respectively) while the red antimicrobial group decreased by 43 % (from 11.6 DID (March 2015) to 7.2 DID (March 2022)) (Fig. 1).

Overall, total antimicrobial consumption in the multilevel model showed consistent correlation with higher DI score (6.6 (95%CI 3.9 to 9.3)) and winter periods (3.6 (95%CI 3.2 to 3.9)). In contrast, before versus during COVID-19 showed a significant reduction in antimicrobial consumption rates (−4.0 (95%CI -4.7 to −3.2)). Both green and red antimicrobial consumption showed similar trends, suggesting that the increase with DI score or winter periods did not favour the consumption of either category. The variance at area level explained 54 %–66 % of the variation in antimicrobial consumption of these models (Table 1). Detail of the individual ATC group level 3 antimicrobial by green and red categories showed similar results (Appendix 5, Appendix 6).

**Table 1:** Multilevel mixed model results of total, green and red categories antimicrobial consumption rates for systemic use (ATC group J01).

Variables	Total AC		Green AC (Preferred)		Red AC (Non-preferred)	
	Coef.	95 % CI	Coef.	95 % CI	Coef.	95 % CI
Deprivation (DI) score	6.56**	(3.85–9.27)	2.76**	(1.47–4.05)	3.75**	(2.27–5.24)
COVID period	−3.96**	(-4.69 to −3.23)	−1.25**	(-1.63 to −0.87)	−2.70**	(-3.19 to −2.22)
Time (months)	−0.03**	(-0.03 to −0.01)	0.018**	(0.011–0.025)	−0.044**	(-0.053 to −0.035)
Winter period	3.56**	(3.21–3.91)	1.80**	(1.60–1.99)	1.57**	(1.38–1.76)
Constant	35.77**	(25.46–46.08)	−2.79	(-8.07–2.49)	38.99**	(32.20–45.78)
Random-effects Parameters (Area)						
var(constant term)	31.22	(18.54–52.57)	7.07	(4.19–11.91)	9.29	(5.49–15.73)
Residual: AR(1)						
rho	0.35	(0.31–0.39)	0.19	(0.15–0.23)	0.53	(0.50–0.57)
var(e)	16.44	(15.46–17.48)	5.9	(5.58–6.23)	5.53	(5.13–5.96)
ICC (Area)***	0.66		0.54		0.64	

ATC: anatomical therapeutic chemical, AC: antimicrobial Consumption, Coef.: coefficient, CI: Confidence interval, ICC: intraclass correlation, AR (1): autoregressive process of order 1.

*p < 0.05, ** p < 0.01.

*** ICC was calculated without residual structures added to the models.

All models were adjusted by gender and under 12 years old population size.

COVID period: January 2020 to March 2022.

Winter period: October to March.

Coefficient interpretation: (1) The between-area interpretation indicates that a difference between two areas of one unit in deprivation score is associated with a difference of 6.56 units in the total of antimicrobial consumption rate (DID). (2) The within-area interpretation indicates that a change within one area of one unit in deprivation score is associated with a change of 6.56 units in the total of antimicrobial consumption rate (DID).

**Fig. 2:**
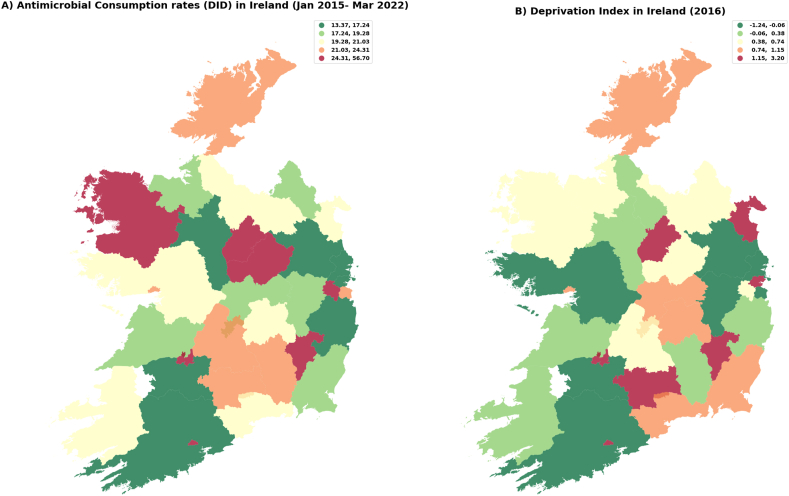
Maps for (2A) antimicrobial consumption rates (in DID) and (2B) deprivation index in Ireland.

Source: elaborated using data provided by the Health Protection and Surveillance Centre, which is based on the antimicrobial sales dataset for Ireland. This dataset encompasses total outpatient antimicrobial consumption, quantified in defined daily doses (DDD) and includes anatomical therapeutic chemical (ATC) group antibacterial agents (J01) intended for systemic use. Antimicrobial consumption (DDD) was standardized using a population-weighted mean, calculated as the ratio resulting in DDD per 1,000 inhabitants per day (DID). Deprivation index (DI) data were obtained from the Department of Public Health & Primary Care at Trinity College Dublin. The colour ranges in the maps are explained in the accompanying boxes, which detail the variations in DID and the deprivation index.

The geographical variation at area level was explored by antimicrobial consumption rates (total, green/red classification and before-during COVID-19) and DI. Fig. 2A shows the antimicrobial consumption rates (in DIDs) map and Fig. 2B deprivation index map. Some areas (Carlow, Longford, Cork City and Limerick City) showed high DI and antimicrobial consumption rates. Other areas, such as Dublin City had high DIs but low antimicrobial consumption rates.

**Fig. 3:**
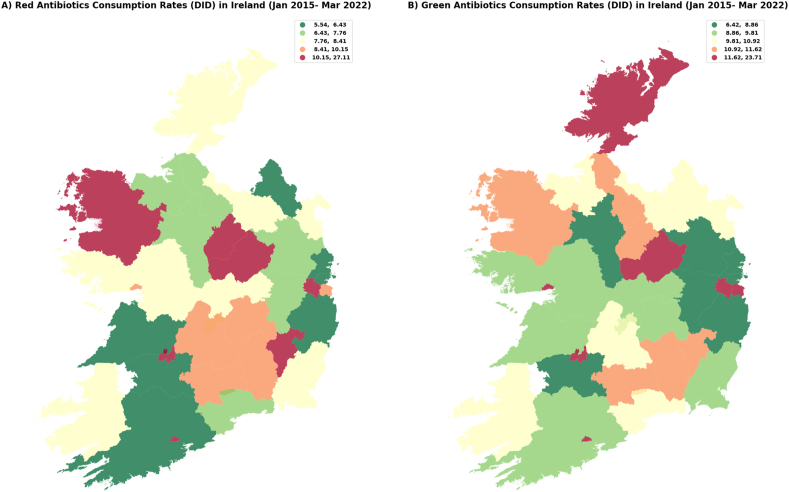
Red (3A) and green (3B) antimicrobial consumption rates (DID) in Ireland (January 2015–March 2022).

Source: elaborated using data provided by the Health Protection and Surveillance Centre, which is based on the antimicrobial sales dataset for Ireland. This dataset encompasses total outpatient antimicrobial consumption, quantified in defined daily doses (DDD) and includes anatomical therapeutic chemical (ATC) group antibacterial agents (J01) intended for systemic use. Antimicrobial consumption (DDD) was standardized using a population-weighted mean, calculated as the ratio resulting in DDD per 1,000 inhabitants per day (DID). Deprivation index (DI) data were obtained from the Department of Public Health & Primary Care at Trinity College Dublin. The colour ranges in the maps are explained in the accompanying boxes, which detail the variations in DID and the deprivation index.

**Fig. 4:**
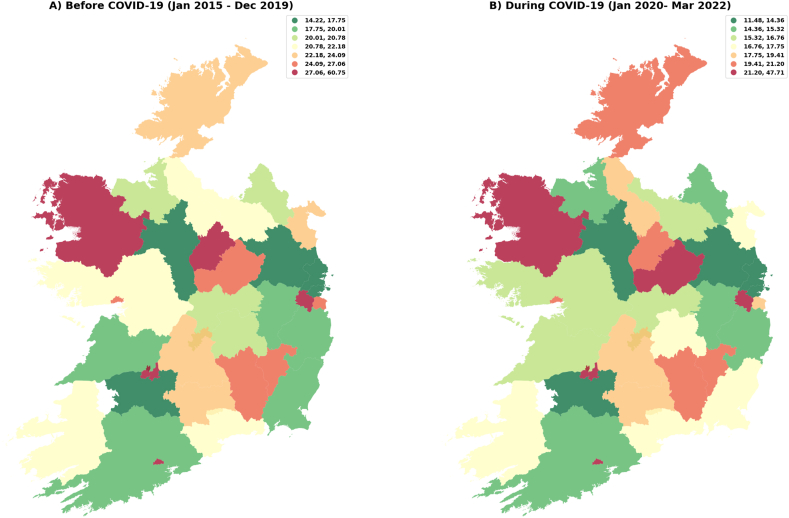
Before (4A) and during (4B) COVID-19 antimicrobial consumption rates (DID) in Ireland (January 2015–March 2022).

Source: elaborated using data provided by the Health Protection and Surveillance Centre, which is based on the antimicrobial sales dataset for Ireland. This dataset encompasses total outpatient antimicrobial consumption, quantified in defined daily doses (DDD) and includes anatomical therapeutic chemical (ATC) group antibacterial agents (J01) intended for systemic use. Antimicrobial consumption (DDD) was standardized using a population-weighted mean, calculated as the ratio resulting in DDD per 1,000 inhabitants per day (DID). Deprivation index (DI) data were obtained from the Department of Public Health & Primary Care at Trinity College Dublin. The colour ranges in the maps are explained in the accompanying boxes, which detail the variations in DID and the deprivation index

Differences in the consumption of red (Fig. 3A) and green (Fig. 3B) antimicrobials (January 2015–March 2022) revealed no consistent trends in antimicrobial consumption were observed. During COVID -19 (Fig. 4A and B), some areas showed slight improvement (green group) in antimicrobial consumption while others showed a reduction in red antimicrobials consumption.

To explore patterns of geographical variation, based on the total antimicrobial consumption rates, global and local spatial autocorrelation tests were carried out. Global Moran's I statistic was −0.049, with a Z-score of −0.25, and a P-value of 0.79 implying a random spatial pattern for consumption rates. Furthermore, the local indicators of spatial correlation (LISA) showed no consistent spatial patterns for consumption rates except for three areas (i.e., Tipperary South, Waterford and Dublin South) (appendices 7 and 8).

## 4 Discussion

A reduction of the antimicrobial consumption rates was observed from January 2015 to March 2022 in Ireland, with an increase in green antimicrobials and a decrease in red antimicrobials. Higher antimicrobial consumption was also observed in more deprived areas and in the winter season. However, a reduction in antimicrobial consumption during COVID-19 was observed but no consistent trends in antimicrobial consumption were observed in relation to deprivation.

This significant relationship between deprivation and antimicrobial consumption found in this analysis is consistent with studies from other countries [[26], [27], [28]]. In Northern Ireland, a comprehensive longitudinal analysis of antimicrobial prescribing focused on primary care found that the overall decrease in antimicrobial prescriptions over time was larger in less deprived areas and significant regional differences were observed in antimicrobial prescribing [26]. Covvey J et al. found an association between increased antimicrobial prescribing rates and deprivation in Scotland from 2010 to 2012 [27]. In England, antimicrobial prescribing was higher in more deprived areas from 2014 to 2018 [28], which was similar to general practice antimicrobial prescribing data from Northern Ireland from 2014 to 2020 [26]. However, socio-economic deprivation is a composite variable that includes individual, contextual and collective determinants, which may not directly affect antimicrobial consumption but act as a proxy for other geographical factors that drive antimicrobial prescribing [8].

The deprivation index serves as a socio-economic indicator that may be shaped by individuals' perceptions and healthcare behaviours, such as self-medication. Factors such as poverty, limited access to healthcare services, low per capita income, and a lack of awareness and literacy have been associated with increased antibiotic use and self-medication [29]. Furthermore, prescribers may be more inclined to provide antibiotics to patients from deprived backgrounds due to concerns about complications, patient pressure and time constraints [30].

Various studies from Europe [31,32], Canada [33] and the United States [34] similarly showed an increase of antimicrobial consumption in the winter months ranging from 21 % to 42 % [[31], [32], [33], [34]]. The reported decrease in antimicrobial consumption during COVID-19 in this study was also reported in France [35], Australia [36], Portugal [37] and Canada [38]. In France, a total reduction of 18 % in prescriptions was observed in 2020, mainly due to a reduction in childrens’ prescriptions and adults≥65 years old [35], while in Australia, a 36 % reduction in antimicrobial sales was reported, particularly in antimicrobials used for respiratory tract infections [36]. Furthermore, in Portugal, monthly reductions in antimicrobial prescriptions/consumption exceeded 45 % especially in April and May 2020 [37], and in Canada, a relative reduction of 37 % in overall outpatient antimicrobial prescriptions was reported [38]. In contrast, Zhong X et al. reported an increase in broad spectrum antimicrobials in response to the pandemic in England (odds ratio 1.37 (95 % CI 1.36 to 1.38) but a subsequent gradual improvement (i.e. decrease) of 1.1–1.2 % per month was observed [39]. In addition, the authors found a consistent pattern showing more deprived areas with higher broad-spectrum antimicrobial prescribing [39].

The observed trends in this study for the consumption of green and red antimicrobials were in line with the Irish National Action Plan (iNAP) on antimicrobial resistance (i.e. encourage green antimicrobials instead of red antimicrobials) [14]. O'Connor et al. reported a 27 % reduction in red antimicrobials in out-of-hours services in Ireland [15]. The presented analysis also showed a correlation between higher deprivation and green and red antimicrobial groups. Further exploration of differences in antimicrobial consumption associated with deprivation could focus on equity and explore its potential wider impact if similar differences are consistently found [40,41].

Our study has several limitations related to data sources. First, the presented analysis was ecological and analysed aggregated data and therefore the results cannot be attributed to individual level hypotheses. Second, antimicrobial consumption data reflects wholesaler-to-retail pharmacy sales and may not reflect true consumption. However, it is comparable to other sources of antimicrobial consumption data used [1]. Third, the DI used in this study was based on the 2016 Irish census data due to the delay in the availability of updated DI (as a result of COVID-19). However, DI scores in Ireland tend to be stable over time and the DI calculated in 2016 is considered a highly representative score [17,42]. Fourth, the lack of consumption rates data for smaller area level (i.e., districts level) led to random spatial patterns rather than showing spatial clusters which did not allow to generalise findings.

European national regulations and policies aim to address AMR by implementing strategies and action plans which includes annual reports on antimicrobial use and resistance surveillance [43]. However, our study suggests that the correlation between deprivation and antimicrobial consumption rates may explain part of the decreasing trend over time, highlighting the need to take into account deprivation when designing and implementing antimicrobial stewardship programs. Local factors such as socioeconomic and health inequalities associated with deprivation should be taken into account as they may influence access to healthcare and prescribing practices of healthcare providers.

## 5 Conclusions

Antimicrobial consumption rates have decreased over time in Ireland. Geographical variations in antimicrobial consumption rates at an area level did not show consistent patterns. However, the correlation between deprivation and antimicrobial consumption rates may explain part of the decreasing trend over time. The findings may have implications for the global fight against AMR and specifically on national regulations and policies on antimicrobial use. Addressing underlying socioeconomic factors contributing to higher antimicrobial consumption in deprived areas is important in the context of reducing AMR in Ireland and other countries.

## Data availability statement

Data is available on request at the HSPC. Data requests are project specific and need to be submitted for each project/analysis.

## Ethical statement

No ethical approval was required as no primary, human material or confidential data was used.

## Funding

This work was funded by grant number RL-20200-03 from Research Leader Awards (RL) 2020, 10.13039/100010414Health Research Board, Ireland and conducted as part of the SPHeRE Programme.

## CRediT authorship contribution statement

Nathaly Garzón-Orjuela*: Conceptualization, Methodology, Software, Validation, Formal analysis, Data Curation, Writing–Original Draft, Visualization. Doaa Amin*: Conceptualization, Methodology, Software, Validation, Formal analysis, Data Curation, Writing–Original Draft, Visualization. **Ajay Oza:** Conceptualization, Writing– Review & Editing, Supervision. **Ricardo Segurado:** Writing– Review & Editing, Supervision. **Akke Vellinga:** Conceptualization, Writing– Review & Editing, Supervision. * These authors contributed equally to this work.

## Declaration of competing interest

The authors declare that they have no known competing financial interests or personal relationships that could have appeared to influence the work reported in this paper.

## Figures and Tables

**Table:** 

Areas	Deprivation score*	Population size of female	Population size of male	Population size of under 12 years old	Latitude	Longitude
Carlow	1.16	28467	28465	9343	−759540.3357	6933174.567
Cavan	0.73	37846	38330	13177	−818528.3007	7169290.26
Clare	0.06	60032	58785	18663	−1006606.826	6953648.233
Cork	−0.41	210258	206953	71809	−983610.7154	6785439.051
Cork City	2.11	63935	61722	13447	−942515.2749	6781350.07
Donegal	1.07	80170	79022	25599	−879887.6703	7347188.051
Dublin (City)	1.75	282284	272270	63488	−695913.9433	7049521.782
Dublin (Dún Laoghaire-Rathdown)	−1.24	113434	104584	30029	−690029.8647	7030392.371
Dublin (Fingal)	−0.36	150780	145240	55451	−696481.7784	7074619.819
Dublin (South)	0.61	142490	136277	48452	−712907.0538	7035083.182
Galway	−0.14	89527	89863	30092	−996394.1181	7050701.974
Galway City	1.10	40868	37800	10033	−1007375.871	7035562.042
Kerry	0.32	74652	73055	21122	−1082246.32	6821982.249
Kildare	−0.21	111958	110546	39878	−759019.7984	7018469.811
Kilkenny	0.27	49699	49533	16033	−805235.0425	6904781.454
Laois	0.79	41886	42811	15567	−818848.547	6980050.135
Leitrim	0.29	15980	16064	5062	−893152.3445	7196930.999
Limerick	−0.20	71803	71642	21770	−965119.5419	6890054.813
Limerick City	3.20	25756	25698	7809	−954301.9086	6921070.418
Longford	1.84	20286	20587	7030	−859936.2194	7115976.581
Louth	1.18	65251	63633	21895	−713995.7552	7153153.623
Mayo	0.40	65460	65047	19333	−1044710.942	7156083.711
Meath	−0.34	98268	96776	36659	−749412.9282	7100794.96
Monaghan	0.61	30520	30866	10505	−770745.1684	7199698.477
Offaly	0.92	39123	38838	12964	−846014.8727	7021633.528
Roscommon	0.13	32167	32377	9997	−918025.0944	7118257.057
Sligo	0.60	33170	32365	9909	−959961.2444	7200144.416
Tipperary North	0.43	35629	35532	11229	−890765.0628	6948386.368
Tipperary South	1.20	44256	44136	13948	−876630.4111	6876205.742
Waterford	1.15	58525	57651	17951	−845680.6683	6831601.956
Westmeath	0.72	44688	44082	14672	−828548.1103	7081446.232
Wexford	0.98	76000	73722	24152	−731487.0102	6883865.152
Wicklow	0.21	72269	70156	24022	−709721.1659	6979055.222

*Negative values imply less deprived and positive values represent areas that are more deprived than the national average.

**Table:** 

ATC group J01	Green (Preferred)	Red (Non-preferred)
Penicillins (J01C)	J01CA01 ampicillin J01CA04 amoxicillin J01CE02 phenoxymethylpenicillin J01CF05 flucloxacillin	J01CR02 amoxicillin and beta-lactamase inhibitor
Cephalosporins and other beta-lactams (J01D)	J01DB01 cefalexin	J01DC02 cefuroxime J01DC04 cefaclor J01DD08 cefixime
Tetracyclines (J01A)	J01AA02 doxycycline J01AA04 lymecycline	J01AA08 minocycline
Macrolides, lincosamides and streptogramis (J01F)	–	J01FA01 erythromycin J01FA09 clarithromycin J01FA10 azithromycin J01FF01 clindamycin
Quinolones (J01M)	–	J01MA01 ofloxacin J01MA02 ciprofloxacin J01MA12 levofloxacin J01MA14 moxifloxacin
Sulfanamides and trimethoprim (J01E)	J01EA01 trimethoprim	–
Other antibacterials (J01X)	J01XE01 nitrofurantoin J01XX01 fosfomycin	–

ATC: anatomical therapeutic chemical.

**Figure:**
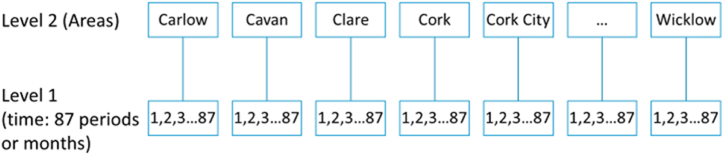


**Table:** 

Variables	Penicillins (J01C)		Cephalosporins and other beta-lactams (J01D)		Tetracyclines (J01A)		Sulfanamides and trimethoprim (J01E)		Other antibacterials (J01X)	
	Coef.	95 % CI	Coef.	95 % CI	Coef.	95 % CI	Coef.	95 % CI	Coef.	95 % CI
Deprivation score	1.42**	(0.77–2.06)	0.11	(-0.01–0.23)	0.66**	(0.27–1.04)	0.25**	(0.12–0.39)	0.31**	(0.12–0.49)
COVID period	−0.98**	(-1.30 to −0.66)	−0.06**	(-0.09 to −0.03)	−0.05	(-0.14–0.04)	−0.06**	(-0.08 to −0.03)	−0.09**	(-0.14 to −0.05)
Time (months)	−0.001	(-0.007–0.004)	0.006**	(0.004–0.006)	0.01**	(0.009–0.013)	−0.002**	(-0.0025 to −0.0015)	0.004**	(0.003–0.005)
Winter period	1.29**	(1.12–1.46)	0.03**	(0.01–0.04)	0.37**	(0.33–0.41)	0.03**	(0.02–0.05)	−0.005	(-0.02–0.01)
Constant	6.21**	(2.01–10.42)	−3.46**	(-3.91 to −3.01)	−5.89**	(-7.25 to −4.53)	2.16**	(1.76–2.56)	−2.00**	(-2.74 to −1.27)
Random-effects Parameters (Area)										
var(constant term)	1.71	(0.99–2.92)	0.06	(0.03–0.11)	0.63	(0.37–1.07)	0.07	(0.04–0.13)	0.15	(0.08–0.24)
Residual: AR(1)										
rho	0.14	(0.10–0.18)	0.5	(0.46–0.53)	0.42	(0.38–0.45)	0.19	(0.15–0.23)	0.66	(0.63–0.69)
var(e)	4.53	(4.29–4.78)	0.02	(0.022–0.025)	0.24	(0.22–0.25)	0.027	(0.026–0.029)	0.05	(0.04–0.06)
ICC (Area) ***	0.27		0.74		0.73		0.74		0.76	

ATC: anatomical therapeutic chemical, Coef.: coefficient, CI: Confidence interval, ICC: intraclass correlation, AR (1): autoregressive process of order 1.

**p < 0.01, *p < 0.05.

*** ICC was calculated without residual structures added to the models.

All models were adjusted by gender and under 12 years old population size.

COVID period: January 2020 to March 2022. Winter period: October to March.

Coefficient interpretation: (1) The between-area interpretation indicates that a difference between two areas of one unit in deprivation score is associated with a difference of 1.42 units in the Penicillins (J01C) consumption rate (DID). (2) The within-area interpretation indicates that a change within one area of one unit in deprivation score is associated with a change of 1.42 units in the Penicillins (J01C) consumption rate (DID).

**Table:** 

Variables	Penicillins (J01C)		Cephalosporins and other beta-lactams (J01D)		Tetracyclines (J01A)		Macrolides, lincosamides and streptogramis (J01F)		Quinolones (J01M)	
	Coef.	95 % CI	Coef.	95 % CI	Coef.	95 % CI	Coef.	95 % CI	Coef.	95 % CI
Deprivation score	1.94**	(1.21–2.67)	0.18**	(0.07–0.28)	0.03	(-0.02–0.08)	1.32**	(0.76–1.89)	0.26**	(0.13–0.40)
COVID period	−1.25**	(-1.47 to −1.03)	−0.28**	(-0.33 to −0.22)	0.008	(-0.02–0.038)	−1.05**	(-1.26 to −0.84)	−0.08**	(-0.12 to −0.04)
Time (months)	−0.019**	(-0.02 to −0.015)	−0.004**	(-0.0054 to −0.0033)	−0.0036**	(-0.004 to −0.0031)	−0.011**	(-0.015 to −0.007)	−0.007**	(-0.008 to −0.006)
Winter period	0.72**	(0.62–0.82)	0.13**	(0.11–0.15)	0.004	(-0.01–0.02)	0.80**	(0.71–0.88)	0.04**	(0.02–0.05)
Constant	16.52**	(13.45–19.59)	3.79**	(3.04–4.55)	2.90**	(2.50–3.30)	11.07**	(8.10–14.03)	5.76**	(5.18–6.34)
Random-effects Parameters (Area)										
var(constant term)	2.27	(1.34–3.83)	0.04	(0.02–0.07)	0.012	(0.007–0.021)	1.33	(0.78–2.26)	0.07	(0.04–0.12)
Residual: AR(1)										
rho	0.38	(0.35–0.42)	0.57	(0.53–0.60)	0.24	(0.20–0.28)	0.54	(0.50–0.57)	0.56	(0.52–0.59)
var(e)	1.41	(1.32–1.50)	0.07	(0.065–0.076)	0.033	(0.031–0.035)	1.08	(1.00–1.16)	0.038	(0.035–0.04)
ICC (Area) ***	0.62		0.40		0.27		0.57		0.66	

ATC: anatomical therapeutic chemical, Coef.: coefficient, CI: Confidence interval, ICC: intraclass correlation, AR (1): autoregressive process of order 1.

**p < 0.01, *p < 0.05.

*** ICC was calculated without residual structures added to the models.

All models were adjusted by gender and under 12 years old population size.

COVID period: January 2020 to March 2022. Winter period: October to March.

Coefficient interpretation: (1) The between-area interpretation indicates that a difference between two areas of one unit in deprivation score is associated with a difference of 1.94 units in the Penicillins (J01C) consumption rate (DID). (2) The within-area interpretation indicates that a change within one area of one unit in deprivation score is associated with a change of 1.94 units in the Penicillins (J01C) consumption rate (DID).

**Figure:**
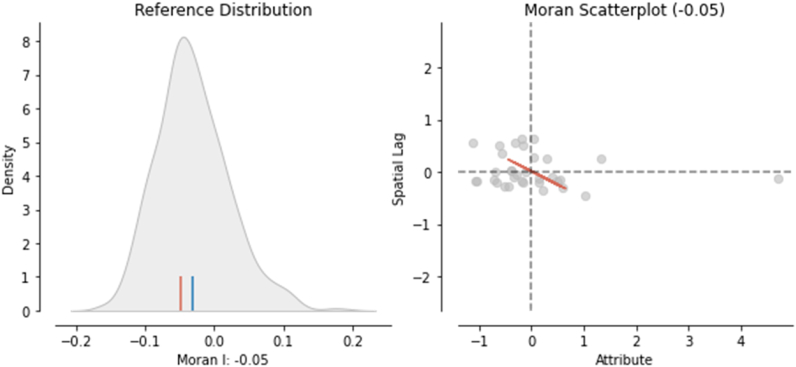


**Figure:**
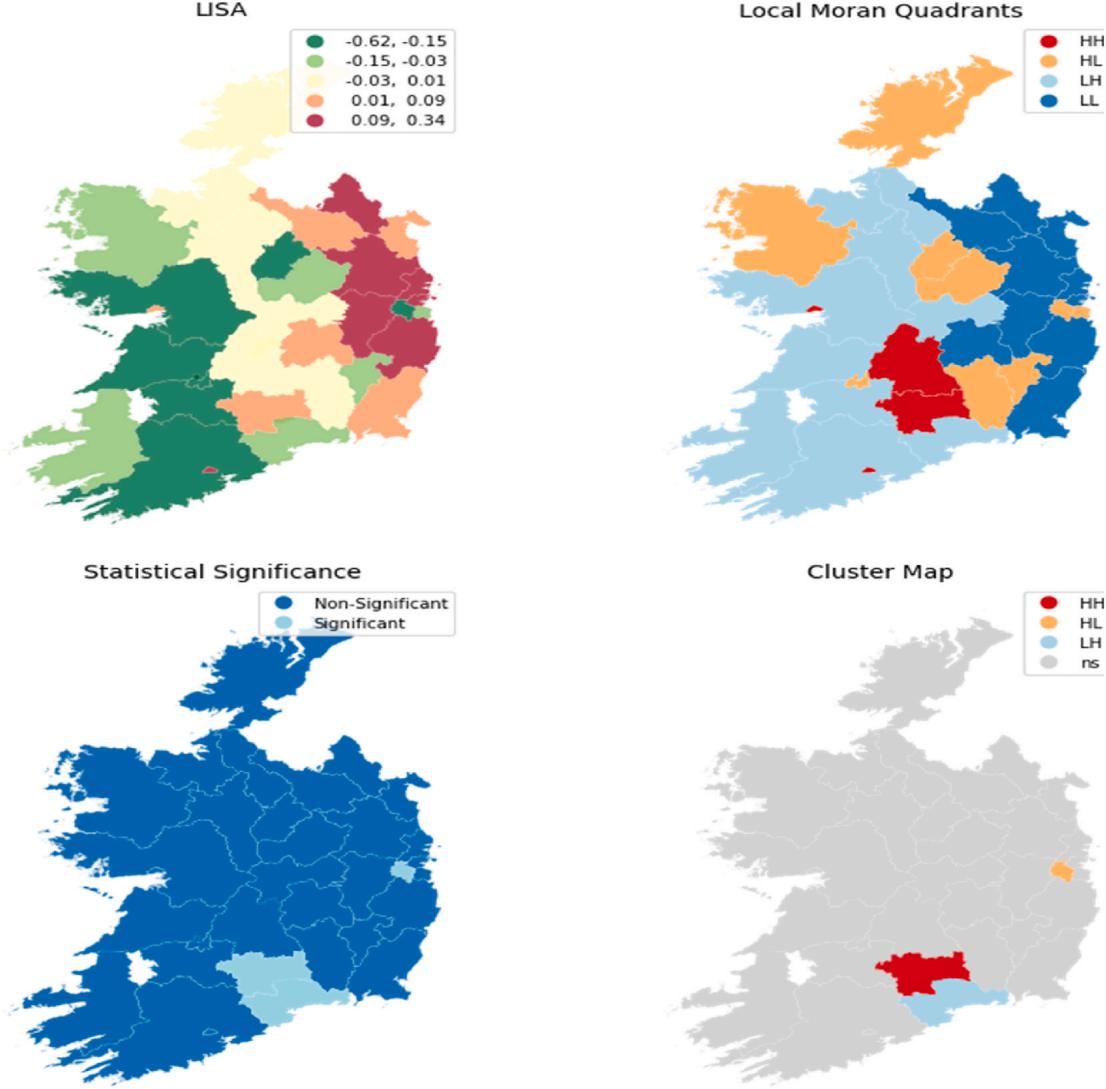


## Appendix 4 Commands for data analysis

In the following link, you can find the code of the analysis: https://github.com/Doaa155/HPSC-Manuscript.git.

## Appendix 8 Local indicators of spatial autocorrelation (LISA)

This figure shows the result of the local indicators of spatial autocorrelation (LISA) test**.** The map to the upper left, showed the values of the local statistic I (LISA). The map on the top right showed the location of LISA on Moran's quadrant plot, which consisted of four quadrants: HH (High-High), HL (High-Low), LL (Low-Low), and LH (Low-High). The LISA quadrant plot reflected cases in which a value of antimicrobial consumption in an aera and the average of its surrounding were more similar, if residing in the HH or LL quadrants, or more dissimilar, if residing in the HL or LH quadrants rather than resulting from complete randomness. Thus, the map to the top right mapped the LISA values of each area based on the quadrant where the LISA resided. The down left map tested the statistical significance based on a threshold value of 5 % and its result showed that only 3 areas (Tipperary South, Waterford, and Dublin South) showed significance and implied that their antimicrobial consumption values were not based on pure chance. The downright map, the cluster map, showed the significant areas that were unlikely to have LISA values resulting from pure randomness mapped to their corresponding quadrant category (HH, or LL, or HL, or LH). Therefore, the LISA test concluded that 3 areas were spatially autocorrelated with their neighbouring areas and the spatial patterns were not based on pure chance.

## References

[1] Antimicrobial Resistance Collaborators (2022). Global burden of bacterial antimicrobial resistance in 2019: a systematic analysis. Lancet.

[2] Aldeyab M.A. (2012). The impact of antibiotic use on the incidence and resistance pattern of extended‐spectrum beta‐lactamase‐producing bacteria in primary and secondary healthcare settings. Br. J. Clin. Pharmacol..

[3] European Centre for Disease Prevention and Control (2023).

[4] HSE/HPSC (2022).

[5] Klein E.Y. (2018). Global increase and geographic convergence in antibiotic consumption between 2000 and 2015. Proc. Natl. Acad. Sci. U. S. A..

[6] Malik B., Bhattacharyya S. (2019). Antibiotic drug-resistance as a complex system driven by socio-economic growth and antibiotic misuse. Sci. Rep..

[7] Curtis H.J., Walker A.J., Mahtani K.R., Goldacre B. (2019). Time trends and geographical variation in prescribing of antibiotics in England 1998-2017. J. Antimicrob. Chemother..

[8] Schmiege D., Evers M., Kistemann T., Falkenberg T. (2020). What drives antibiotic use in the community? A systematic review of determinants in the human outpatient sector. Int. J. Hyg Environ. Health.

[9] Conway R. (2015). Deprivation index and dependency ratio are key determinants of emergency medical admission rates. Eur. J. Intern. Med..

[10] Cournane S. (2015). Social deprivation and hospital admission rates, length of stay and readmissions in emergency medical admissions. Eur. J. Intern. Med..

[11] Madden J.M. (2021). Population mobility trends, deprivation index and the spatio-temporal spread of coronavirus disease 2019 in Ireland. Int. J. Environ. Res. Publ. Health.

[12] Quigley P., Usher C., Bennett K., Feely J. (2006). Socioeconomic influences on benzodiazepine consumption in an Irish Region. Eur. Addiction Res..

[13] (2016). Census of population 2016- profile 2 population distribution and movements. https://www.cso.ie/en/releasesandpublications/ep/p-cp2tc/cp2pdm/bgn/.

[14] Government of I. (2021).

[15] O'Connor N. (2020). Improving the quality of antibiotic prescribing through an educational intervention delivered through the out-of-hours general practice service in Ireland. Eur. J. Gen. Pract..

[16] Trinity National Deprivation Index - School of Medicine - Trinity College Dublin. [cited 2022 25 November]; Available from: https://www.tcd.ie/medicine/public_health_primary_care/research/deprivation/.

[17] Teljeur C., Darker C., Barry J., O’Dowd T. (2019). https://www.drugsandalcohol.ie/34675/.

[18] Central Statistics Office. [cited 2022 25 November]; Available from: https://data.gov.ie/organization/central-statistics-office?tags=census-2016.

[19] Persons in Communal Establishments, Administrative County, Census 2016, Theme 7.1, Ireland, 2016, CSO & OSi | Persons in Communal Establishments, Administrative County, Census 2016, Theme 7.1, Ireland, 2016, CSO & OSi | OSi Census 2016 OpenData. [cited 25 November 2022]; Available from: https://census2016.geohive.ie/datasets/813ec01e603d4fc1bc4cf292007adb6a_0/explore?location=53.378053%2C-8.330000%2C7.82.

[20] Census 2011 Boundary Files - CSO - Central Statistics Office. [cited 2022 25 November]; Available from: https://www.cso.ie/en/census/census2011boundaryfiles/.

[21] Eurostat - Data Explorer. [cited 2022 25 November]; Available from: https://appsso.eurostat.ec.europa.eu/nui/show.do?dataset=demo_pjan&lang=en.

[22] Steele F. (2014). http://www.bristol.ac.uk/cmm/learning/course.html.

[23] Twisk J.W.R. (2013).

[24] Stata B. (2021). https://www.stata.com/bookstore/multilevel-mixed-effects-reference-manual/.

[25] Stata B. (2021). https://www.stata.com/bookstore/time-series-reference-manual/.

[26] Devine P., O'Kane M., Bucholc M. (2021). Trends, variation, and factors influencing antibiotic prescribing: a longitudinal study in primary care using a multilevel modelling approach. Antibiotics.

[27] Covvey J.R. (2014). An association between socioeconomic deprivation and primary care antibiotic prescribing in Scotland. J. Antimicrob. Chemother..

[28] Thomson K. (2020). An examination of trends in antibiotic prescribing in primary care and the association with area-level deprivation in England. BMC Publ. Health.

[29] Malik B., Hasan Farooqui H., Bhattacharyya S. (2022). Disparity in socio-economic status explains the pattern of self-medication of antibiotics in India: understanding from game-theoretic perspective. R. Soc. Open Sci..

[30] Adekanmbi V., Jones H., Farewell D., Francis N.A. (2020). Antibiotic use and deprivation: an analysis of Welsh primary care antibiotic prescribing data by socioeconomic status. J. Antimicrob. Chemother..

[31] Achermann R. (2011). Antibiotic use in adult outpatients in Switzerland in relation to regions, seasonality and point of care tests. Clin. Microbiol. Infect..

[32] Elseviers M.M., Ferech M., Vander Stichele R.H., Goossens H. (2007). Antibiotic use in ambulatory care in Europe (ESAC data 1997-2002): trends, regional differences and seasonal fluctuations. Pharmacoepidemiol. Drug Saf..

[33] Patrick D.M. (2004). Per capita antibiotic consumption: how does a North American jurisdiction compare with Europe?. Clin. Infect. Dis..

[34] Suda K.J. (2014). Trends and seasonal variation in outpatient antibiotic prescription rates in the United States, 2006 to 2010. Antimicrob. Agents Chemother..

[35] Bara W., Brun-Buisson C., Coignard B., Watier L. (2022). Outpatient antibiotic prescriptions in France: patients and providers characteristics and impact of the COVID-19 pandemic. Antibiotics.

[36] Gillies M.B. (2022). Changes in antibiotic prescribing following COVID-19 restrictions: lessons for post-pandemic antibiotic stewardship. Br. J. Clin. Pharmacol..

[37] Silva T.M. (2021). The impact of the COVID-19 pandemic on antibiotic prescribing trends in outpatient care: a nationwide, quasi-experimental approach. Antibiotics.

[38] Kitano T. (2021). The impact of COVID-19 on outpatient antibiotic prescriptions in Ontario, Canada; an interrupted time series analysis. Open Forum Infect. Dis..

[39] Zhong X. (2023 May 16). Impact of COVID-19 on broad-spectrum antibiotic prescribing for common infections in primary care in England: a time-series analyses using OpenSAFELY and effects of predictors including deprivation. Lancet Reg Health Eur.

[40] Eslava-Schmalbach J. (2019). Conceptual framework of equity-focused implementation research for health programs (EquIR). Int. J. Equity Health.

[41] Moran D. (2019). A framework for improved one health governance and policy making for antimicrobial use. BMJ Glob. Health.

[42] Haase T., Pratschke J. (2005). Deprivation and its patial Articulation in the Republic of Ireland: New Measures of Deprivation Based on the Census of Population, 1991, 1996 and 2002.

[43] European Centre for Disease Prevention and Control. Strategies and action plans on antimicrobial resistance. [cited 2023 December]; Available from: https://www.ecdc.europa.eu/en/publications-data/directory-guidance-prevention-and-control/antimicrobial-resistance-strategies.

